# A comparative study on the efficacy, safety, and cost-effectiveness of bimatoprost/timolol and dorzolamide/timolol combinations in glaucoma patients

**DOI:** 10.4103/0253-7613.71917

**Published:** 2010-12

**Authors:** R. Jothi, A.M. Ismail, R. Senthamarai, Siddhartha Pal

**Affiliations:** RVS College of Pharmaceutical Sciences, Sulur, Coimbatore-641 402, Tamilnadu, India; 1 Department of Pharmacy Practice, Periyar College of Pharmaceutical Sciences for Girls, Trichirappalli-620 021, Tamilnadu, India

**Keywords:** Bimatoprost/timolol, dorzolamide/timolol, intraocular pressure, ocular hypertension

## Abstract

**Aim:**

This study was designed to compare the bimatoprost/timolol combination and dorzolamide/timolol combination in glaucoma for efficacy, safety, and cost-effectiveness in a local population of Trichy in the state of Tamilnadu.

**Materials and Methods:**

Eight-week, randomized, parallel group, open-label study was conducted on 48 patients of open angle glaucoma or ocular hypertension. After initial clinical assessment and baseline investigations, bimatoprost/timolol combination (Group A) was prescribed to 22 patients (2 patients lost after initial assessment) and dorzolamide/timolol combination (Group B) to 24 patients. The patients were reviewed after second and eighth weeks for cure rate and adverse drug reaction monitoring.

**Results:**

At the end of 8 weeks, the mean reduction in intraocular pressure from baseline was 13.04 mmHg (95% confidence interval (CI): 10.67–14.70) with bimatoprost/timolol combination once daily (*P* < 0.01) and 9.46 mmHg (95% CI: 7.47–10.5) with dorzolamide/timolol combination twice daily. Both the treatments were safe. Cost-effective range of bimatoprost/timolol combination was lower than that of dorzolamide/timolol combination.

**Conclusion:**

The fixed combination of bimatoprost/timolol was slightly more effective than that of dorzolamide/timolol combination in reducing IOP, and both treatments were generally well tolerated. Bimatoprost/timolol combination was more cost-effective (cost-effective analysis) than dorzolamide/timolol combination.

## Introduction

Glaucoma affects nearly 66.7 million individual worldwide, and it is one of the leading causes of irreversible blindness.[1,2] Glaucoma is a group of ocular disorder involving optic neuropathy characterized by changes in optic nerve head and loss of visual sensitivity and field.[3] It is estimated that the number of patients with glaucoma will increase up to 79.6 million by the year 2020. Of these, 74% will have open angle glaucoma. From 2010 to 2020, the most detectable change in glaucoma worldwide will be an increase of the incidence of glaucoma in India.[4,5]

Lowering the intraocular pressure (IOP) by early treatment reduces the loss of visual field in patients with glaucoma and ocular hypertension.[6,7] When a single medication does not reduce IOP significantly, a fixed combination may improve both the compliance and the quality of life.[8] A fixed dose combination of latanoprost and timolol was first prostaglandin analogue/beta-blocker made available in the market. Bimatoprost/timolol is a new fixed combination antiglaucoma therapy. Among the newer agents available to treat glaucoma and ocular hypertension are prostaglandin analogues, carbonic anhydrase inhibitors, and beta blockers.[7] Comparison studies have been done between these group of drugs.[1,9–12] In this study, the effect of bimatoprost/timolol has been compared with that of dorzolamide/timolol in glaucoma patients in India.

## Materials and Methods

### Study design

This interventional, 8-week, randomized, open-label, parallel-group study was conducted at an eye hospital in Trichy city. The study was carried out from June to December 2008. The protocol was approved by Institutional Ethics Committee (IEC). Informed consent was obtained from patients prior to the conduct of the study.

### Patient selection

Inclusion/exclusion criteria are mentioned below. Patients on taking ocular hypotensive medication underwent the following washout periods before inclusion: 4 days for parasympathomimetics or carbonic anhydrase inhibitors, 2 weeks for sympathomimetics or topical alpha adrenergic agonists, 4 weeks for topical alpha blockers, prostaglandin, and combination therapy.

### Inclusion criteria

Following patients were included in the study:

- Age: 21 years or more
- Patients with chronic open angle glaucoma, ocular hypertension, pseudoexfoliative glaucoma, and pigmentary glaucoma.
- IOP in each eye ≥22 mmHg at 8 a.m. on day 0 (after washout)
- Best corrected visual acuity 20/100 or better in each eye

### Exclusion criteria

Patients with anticipated alteration of ongoing therapy with agents that could interact with study medications, chronic use of ocular medication other than the study drugs, progressive or functionally significant visual field loss within the past year, pregnant or lactating females, patients with dry eyes, corneal abnormality or any other conditions that prevent related applanation tonometry, patients with ocular infection, advanced cataract, ocular inflammation or history of renal, or hepatic impairment were excluded from the study.

### Study visits and treatment schedule

On the basis of the inclusion and exclusion criteria, 48 patients were selected during the study period. At baseline visit apart from the personal characteristics, IOP was measured using applanation tonometry (Gem Optical Instruments Industry, Haryana, India). The patients were randomized into two groups (according to their economic status) to receive either fixed combination of bimatoprost/timolol once a day (evening) (Group A) or dorzolamide/timolol twice a day (Group B). Initial readings were considered as baseline, first review values were taken at the end of second week and the second review values at the end of eighth week. Participants on baseline were advised to instill the medication according to the treatment regimen. The participants were recalled at second and eighth weeks for follow-up evaluation. The baseline assessment included IOP, visual acuity (decimal value), pupil size, heart rate, and blood pressure. These assessments were also done at the end of second and eighth week visits. Adverse drugs reactions (ADRs) reported by the patients were noted.

### Intervention

Following interventions were carried out:

1. Bimatoprost/timolol solution to be applied once daily at the rate of one drop in the affected eye/eyes. Each 3 mL medication bottle containing bimatoprost 0.03% and timolol 0.5% solution was priced at Rs. 405. One drop of the solution contained 0.015 mg of bimatoprost and 0.25 mg of timolol.
2. Dorzolamide/timolol solution was applied twice daily at the rate of one drop in the affected eye/eyes. Each medication bottle contained 5 mL solution of dorzolamide 2% and timolol 0.5% and was priced at Rs. 235.50. One drop of solution contained 1 mg of dorzolamide and 0.25 mg of timolol.

### Pharmacoeconomic analysis

Main types of cost analysis include cost-of-illness analysis, cost-minimization analysis, cost-effectiveness analysis (CEA), cost-utility analysis (CUA), cost-consequence analysis, and cost-benefit analysis (CBA).

### Cost-effectiveness analysis[13]

The cost effectiveness was calculated, and the two interventions were compared on the basis of amount needed to treat the patient. Initially for both medications, one day cost was calculated. Then, the annual cost was calculated. Annual cost was divided by minimum IOP reduction value (mmHg) and maximum IOP reduction value (mmHg) to find the cost-effective range for both combinations.

### Statistics

The values for visual acuity, heart rate, pupil size, systolic blood pressure, diastolic blood pressure, and IOP between treatment groups were statistically evaluated by a repeated measures analysis of variance (ANOVA). All the values at individual time point were evaluated by intragroup comparisons made between the values obtained under baseline and treatment conditions. For this purpose, Dunnett’s multiple comparison test was used. Statistical significance was achieved with *P* ≤ 0.05. Adverse events were evaluated by Fisher Exact test.

## Results

The 8-week study was completed by 22 out of 24 (91.67%) patients in bimatoprost/timolol group, and 24 out of 24 (100%) patients in dorzolamide/timolol group. Two patients were lost to follow-up in group A. The characteristics of patients included in this study are given in Table 1.

**Table 1:** Patient characteristics (n = 46)

*Characteristics*	*Drugs*	
	*Bimatoprost / timolol*	*Dorzolamide / timolol*
Number of patients	22	24
Age (years)	58.95 ± 10.62	62.42 ± 8.29
Gender		
Male (%)	13 (59)	15 (62)
Family history of glaucoma (%)	3 (14)	2 (8)
Diagnosis of study		
POAG (%)	22 (100)	19 (79)
OHT (%)	–	2 (8)
Pseudoexfoliative glaucoma (%)	–	3 (13)
Medical history		
Hypertension (%)	4 (18)	2 (8)
Diabetes mellitus (%)	7 (32)	9 (38)
Thyroid disorder (%)	1 (5)	–
Myocardial infarction (%)	2 (9)	–
Diabetes mellitus/Hypertension (%)	1 (5)	4 (17)
Social History		
Smoker or alcoholics (%)		
	12 (55)	13 (54)

POAG = Primary open angle glaucoma; OHT = ocular hypertension

### Efficacy and safety

The mean changes in visual acuity, heart rate, pupillary size, and systolic/diastolic blood pressure were insignificant in both groups. Gradual reduction of IOP in both the groups was seen [Table 2]. Reduction was higher with bimatoprost/timolol combination than that of dorzolamide/timolol. These reductions for both the groups are significant at both the review phases. The 95% confidence interval for both group A and group B patients were 10.45–14.91 and 7.51–10.50, respectively. Both regimens were safe and well tolerated. No serious treatment-related adverse events were reported. Most of the adverse drug reactions were ocular or periocular and mild in severity [Table 3]. Bimatoprost/timolol combination was found to be more cost-effective than dorzolamide/timolol combination [Table 4].

**Table 2:** Changes in intraocular pressure (IOP)

*Parameters*	*Group A (Bimatoprost/timolol)*			*Group B (Dorzolamide/timolol)*		
	*Baseline*	*First review*	*Second review*	*Baseline*	*First review*	*Second review*
IOP (mean ± SD)	28.49 ± 5.33	20.00 ± 0.28*	15.45 ± 2.26*	26.71 ± 4.60	20.90 ± 3.10*	17.25 ± 1.85*

* *P* < 0.01 in comparison to baseline value (ANOVA and Dunnett’s multiple comparison tests).

**Table 3:** Adverse drug reactions

*Adverse drug reaction*	*% Patients (n = 46)*	
	*Bimatoprost/timolol combination (Group A)*	*Dorzolamide/timolol combination (Group B)*
Eye irritation	2	2
Ocular burning/	6	12
stinging		
Headache	1	–
Disorder of taste	2	4
Conjunctival	1	–
hyperemia		
Foreign body	–	1
sensation		
Redness of eye	4	1
Browache	1	1

**Table 4:** Cost-effectiveness analysis of bimatoprost/timolol and dorzolamide/timolol combinations (n = 46)

*Treatment*	*Drops per bottle*	*Days per bottle*	*Annual usage (bottles per year)*	*Bottle cost (Rs. per bottle)*	*Annual Cost*	*Minimum IOP reduction, value (mmHg)*	*Maximum IOP reduction, value (mmHg)*	*Cost-effectiveness range (Rs/mmHg decrease in IOP)*	
Bimatoprost/timolol combination	60	30	12.1	Rs. 405	Rs. 4900.50	6	23	Rs. 213.93–Rs. 817.9	
Dorzolamide/timolol combination	100	25	14.6	Rs. 235.50	Rs. 3438.30	3	19	Rs. 180.94–Rs. 1146	

IOP = Intraocular pressure.

## Discussion

In this study, IOP level was reduced significantly with both bimatoprost/timolol as well as dorzolamide/timolol combination. However, bimatoprost/timolol combination showed greater effectiveness than that of dorzolamide/timolol. Both these combinations had no effect on heart rate, blood pressure, and pupil size. The adverse drug reactions of bimatoprost/timolol were less than the dorzolamide/timolol. Previous pharmacoeconomic studies have concluded that bimatoprost is a cost-effective drug.[14] However, in our study both the combinations were compared and on pharmacoeconomic analysis it was found that bimatoprost/timolol combination was more cost-effective.
